# Supplementary material for: Smooth Interpolating Curves with Local Control and Monotone Alternating Curvature
Source: Comput Graph Forum. 2022 Oct 6;41(5):25–38. doi: 10.1111/cgf.14600 (PMC9827861; doi:10.1111/cgf.14600)
Supplement: Supplementary file 1 — Supplement Material [file CGF-41-25-s001.zip › Local-Smooth-Interpolating-MonoCurvature/extern/clothoids/submodules/Utils/ThirdParties/fmt/fmt-8.1.1.zip!/fmt-8.1.1/doc/html/index.html]

Overview — fmt 8.1.1 documentation


Toggle navigation

{fmt}

- 8.1.1
  - 8.1.1
  - 8.1.0
  - 8.0.1
- Contents
- Usage
- API
- Syntax

# {fmt}

A modern formatting library

Download


- Version 8.1.1
- Version 8.1.0
- Version 8.0.1

# Overview¶

**{fmt}** is an open-source formatting library providing a fast and safe
alternative to C stdio and C++ iostreams.

What users say:

Thanks for creating this library. It’s been a hole in C++ for
a long time. I’ve used both `boost::format` and
`loki::SPrintf`, and neither felt like the right answer.
This does.

## Format API¶

The format API is similar in spirit to the C `printf` family of function but
is safer, simpler and several times faster
than common standard library implementations.
The format string syntax is similar to the one used by
str.format in
Python:

```
std::string s = fmt::format("The answer is {}.", 42);
```

The `fmt::format` function returns a string “The answer is 42.”. You can use
`fmt::memory_buffer` to avoid constructing `std::string`:

```
auto out = fmt::memory_buffer();
format_to(std::back_inserter(out),
          "For a moment, {} happened.", "nothing");
auto data = out.data(); // pointer to the formatted data
auto size = out.size(); // size of the formatted data
```

The `fmt::print` function performs formatting and writes the result to a stream:

```
fmt::print(stderr, "System error code = {}\n", errno);
```

If you omit the file argument the function will print to `stdout`:

```
fmt::print("Don't {}\n", "panic");
```

The format API also supports positional arguments useful for localization:

```
fmt::print("I'd rather be {1} than {0}.", "right", "happy");
```

You can pass named arguments with `fmt::arg`:

```
fmt::print("Hello, {name}! The answer is {number}. Goodbye, {name}.",
           fmt::arg("name", "World"), fmt::arg("number", 42));
```

If your compiler supports C++11 user-defined literals, the suffix `_a` offers
an alternative, slightly terser syntax for named arguments:

```
using namespace fmt::literals;
fmt::print("Hello, {name}! The answer is {number}. Goodbye, {name}.",
           "name"_a="World", "number"_a=42);
```

## Safety¶

The library is fully type safe, automatic memory management prevents buffer
overflow, errors in format strings are reported using exceptions or at compile
time. For example, the code

```
fmt::format("The answer is {:d}", "forty-two");
```

throws the `format_error` exception because the argument `"forty-two"` is a
string while the format code `d` only applies to integers.

The code

```
format(FMT_STRING("The answer is {:d}"), "forty-two");
```

reports a compile-time error on compilers that support relaxed `constexpr`.
See here for details.

The following code

```
fmt::format("Cyrillic letter {}", L'\x42e');
```

produces a compile-time error because wide character `L'\x42e'` cannot be
formatted into a narrow string. For comparison, writing a wide character to
`std::ostream` results in its numeric value being written to the stream
(i.e. 1070 instead of letter ‘ю’ which is represented by `L'\x42e'` if we
use Unicode) which is rarely desirable.

## Compact Binary Code¶

The library produces compact per-call compiled code. For example
(godbolt),

```
#include <fmt/core.h>

int main() {
  fmt::print("The answer is {}.", 42);
}
```

compiles to just

```
main: # @main
  sub rsp, 24
  mov qword ptr [rsp], 42
  mov rcx, rsp
  mov edi, offset .L.str
  mov esi, 17
  mov edx, 1
  call fmt::v7::vprint(fmt::v7::basic_string_view<char>, fmt::v7::format_args)
  xor eax, eax
  add rsp, 24
  ret
.L.str:
  .asciz "The answer is {}."
```

## Portability¶

The library is highly portable and relies only on a small set of C++11 features:

- variadic templates
- type traits
- rvalue references
- decltype
- trailing return types
- deleted functions
- alias templates

These are available in GCC 4.8, Clang 3.4, MSVC 19.0 (2015) and more recent
compiler version. For older compilers use {fmt} version 4.x which is maintained and
only requires C++98.

The output of all formatting functions is consistent across platforms.
For example,

```
fmt::print("{}", std::numeric_limits<double>::infinity());
```

always prints `inf` while the output of `printf` is platform-dependent.

## Ease of Use¶

{fmt} has a small self-contained code base with the core library consisting of
just three header files and no external dependencies.
A permissive MIT license allows
using the library both in open-source and commercial projects.

Learn more…

GitHub Repository

© Copyright 2012-present, Victor Zverovich.
Created using Sphinx 3.3.0.
